# Bridging the gap between biomarkers and bedside decisions: the prognostic value of the blood urea nitrogen-to-albumin ratio in sepsis-associated AKI

**DOI:** 10.3389/fnut.2026.1831995

**Published:** 2026-08-13

**Authors:** Hongmin Chen, Xiaoxiao Qu, Xinxing Wang, Guosong Jiang, Zimiao Zhu

**Affiliations:** 1 Department of Clinical Laboratory, Wenzhou Longwan Hospital of Integrated Traditional Chinese and Western Medicine, Wenzhou, Zhejiang, China; 2 Department of Clinical Laboratory, The Second Affiliated Hospital and Yuying Children's Hospital of Wenzhou Medical University, Wenzhou, Zhejiang, China; 3 Department of Clinical Laboratory, Chengdu BOE Hospital, Chengdu, Sichuan, China; 4 Department of Pulmonary and Critical Care Medicine, The First People's Hospital of Zhaotong and Zhaotong Hospital Affiliated to Kunming Medical University, Zhaotong, Yunnan, China; 5 Department of Clinical Laboratory, Central Blood Station of Wenzhou, Wenzhou, Zhejiang, China

**Keywords:** 28-day mortality, blood urea nitrogen-to-albumin ratio (BAR), continuous renal replacement therapy (CRRT), prognostic stratification, sepsis-associated AKI, subgroup analysis

## Abstract

**Background:**

While sepsis-associated acute kidney injury (AKI) is a leading cause of mortality in intensive care units, identifying high-risk patients remains clinically challenging. Traditional severity scores often fail to capture dynamic metabolic disturbances. This study aimed to characterize the association between the blood urea nitrogen-to-albumin ratio (BAR)—a novel biomarker reflecting systemic nitrogen metabolism and nutritional status—and 28-day mortality in patients with sepsis-associated AKI undergoing continuous renal replacement therapy (CRRT).

**Methods:**

This prospective observational study included 790 patients with sepsis-associated AKI undergoing CRRT. log_2_BAR was evaluated as a candidate prognostic variable. Multivariable Cox proportional hazard regression models were used to determine the association with 28-day mortality. The dose–response relationship was characterized by restricted cubic splines (RCS). The prognostic accuracy and incremental predictive value of log_2_BAR beyond traditional risk models were assessed using the area under the receiver operating characteristic curve (AUC), net reclassification improvement (NRI), and integrated discrimination improvement (IDI). Calibration and clinical utility were further evaluated using calibration curves and decision curve analysis (DCA). Model robustness was validated through subgroup analyses, categorical stratification, and E-value quantification to assess potential unmeasured confounding.

**Results:**

Multivariable analysis identified log_2_BAR as an independent predictor of 28-day mortality (adjusted HR = 1.33, 95% CI: 1.15~1.53, *P* < 0.001). RCS analysis revealed a significant dose–response relationship, with mortality risk increasing monotonically with log_2_BAR. The integration of log_2_BAR into traditional risk models significantly improved prognostic accuracy (AUC increased from 0.772 to 0.783, *P* < 0.05). Sensitivity analyses, including categorical stratification and E-value calculations, confirmed the robustness of these findings against potential unmeasured confounders across different Modelspecifications and population subsets.

**Conclusions:**

log_2_BAR is a robust and independent predictor of 28-day mortality in patients with sepsis-associated AKI treated with CRRT. Integrating this readily available biomarker provides valuable prognostic precision, supporting its use as a supplementary prognostic tool for early risk stratification in critically ill patients.

## Introduction

Sepsis-associated acute kidney injury (AKI) remains a critical clinical challenge in intensive care units, contributing significantly to high morbidity and mortality rates (1). In China, the incidence of sepsis-associated AKI has risen sharply, becoming a primary driver of renal replacement therapy (RRT) requirements and long-term adverse outcomes (2, 3). The blood urea nitrogen-to-albumin ratio (BAR), an integrated biomarker reflecting both systemic nitrogen metabolism and nutritional status, has recently emerged as a potential prognostic indicator in critical care medicine (4–7). Given the synergistic impact of systemic inflammation and protein catabolism in these patients, early identification of high-risk individuals is critical to optimizing clinical management and improving survival.

Although the prognostic value of BAR has been explored in various cardiovascular and critical care settings, its specific utility in the sepsis-associated AKI population warrants independent investigation due to their fundamentally different metabolic profiles (4, 8, 9). Unlike general ICU populations, where AKI is often transient, patients with sepsis-associated AKI undergoing continuous renal replacement therapy (CRRT) exhibit profound metabolic derangements, characterized by high rates of muscle wasting, “overflow” of urea, and severe systemic inflammation (10–13). These factors lead to a higher baseline of BUN and a lower baseline of albumin, suggesting that the “standard” BAR reference ranges derived from general cohorts may lead to significant misclassification. Consequently, it remains unclear whether BAR remains a sensitive “value-added” biomarker or if its diagnostic efficiency is blunted by the overall metabolic derangement inherent in sepsis.

To address this gap, we utilized a clinical cohort of patients with sepsis-associated AKI treated with CRRT to investigate the prognostic value of log_2_BAR specifically within this high-risk metabolic context. This study aims to: Evaluate the independent association between log_2_BAR and 28-day mortality using multivariable Cox regression; Characterize the dose-response relationship between log_2_BAR and mortality risk using restricted cubic spline (RCS) analysis; Assess the incremental predictive value of log_2_BAR beyond traditional risk models through ROC, NRI, and IDI;Furthermore, assess Modelcalibration using Calibration Curves (CAL) to verify the agreement between predicted and observed risks, and employ Decision Curve Analysis (DCA) to quantify the net clinical benefit across varied intervention thresholds. Validate the stability of the findings through rigorous subgroup and sensitivity analyses (including tertiles and quintiles); and Quantify the robustness of the association against unmeasured confounding using E-values. By establishing population-specific prognostic insights, this study provides a more nuanced, non-invasive tool for early risk stratification in patients with sepsis-associated AKI.

## Methods

### Study design and data source

This study was a secondary analysis of a publicly available, de-identified dataset originally provided by Seung Hyeok Han from the Department of Internal Medicine, College of Medicine, Institute of Kidney Disease Research, Yonsei University, Seoul, Korea. The dataset was obtained from the Dryad Digital Repository (https://doi.org/10.5061/dryad.6v0j9) and includes patients admitted to the intensive care units (ICU) of Yonsei University Health System Severance Hospital and the National Health Insurance Service Medical Center Ilsan Hospital between January 2009 and September 2016 (14). All patients received continuous renal replacement therapy (CRRT). As this study utilizes a pre-defined, publicly available dataset, the study period and institutional setting were inherently fixed. To bridge the gap between the dataset's historical timeframe and current clinical standards, we implemented a rigorous *post-hoc* screening protocol based on Sepsis-3 definitions and focused on a subset of patients receiving a consistent, high-intensity CRRT dose. This approach ensures that our evaluation of the log_2_BAR index is methodologically standardized .

### Study population

The study population comprised patients admitted to the ICUs of the participating institutions between January 2009 and September 2016. We initially identified (*n* = 2,391) patients who received continuous renal replacement therapy (CRRT) during their ICU stay. To ensure the clinical relevance and diagnostic accuracy of our cohort, we applied the criteria established by the Third International Consensus Definitions for Sepsis and Septic Shock (Sepsis-3) (15, 16). The study population was strictly selected based on the following inclusion and exclusion criteria:Inclusion Criteria: (1). A diagnosis of sepsis, defined as a suspected or documented infection with an acute increase in the Sequential Organ Failure Assessment (SOFA) score of ≥2 points from baseline. (2). Acute kidney injury (AKI) stage 2 or 3, classified according to the Acute Kidney Injury Network (AKIN) criteria. (3). Requirement for CRRT during the ICU admission.Exclusion Criteria: (1). Age < 18 years. (2). Pre-existing chronic kidney disease (CKD) or a history of end-stage renal disease. (3). Prior dialysis or CRRT treatment before the current admission. (4). Pregnancy, postrenal obstruction, or a history of kidney transplantation.After applying these criteria, a final cohort of 790 patients with sepsis-associated AKI who received CRRT was included in the final analysis (Supplementary Figure 1).

### CRRT protocol

Nephrologists decided whether or not to initiate CRRT upon the development of sepsis with AKI in ICU patients. The indications for CRRT use included uncontrolled volume overload, intractable hyperkalaemia or metabolic acidosis. The type of CRRT used was continuous venovenous haemodiafiltration through the internal jugular, subclavian or femoral veins. CRRT was started at a blood flow rate of 100 ml/min and was increased up to 150 ml/min (17, 18).The total effluent volume as a sum of the dialysis and replacement doses was targeted to deliver ≥35 ml/kg per h in all patients using a multiFiltrate machine (Fresenius Medical Care, Bad Homburg, Germany) or a PRISMAFLEX System (Baxter International, Lund, Sweden).

### Data collection

Clinical data were extracted from the electronic medical records of the participating hospitals. Collected variables included: Baseline demographics: Age, sex, and body mass index (BMI).Comorbidities: History of diabetes mellitus, hypertension, cerebrovascular disease, and chronic heart failure, quantified using the Charlson Comorbidity Index (CCI).Laboratory test results: Blood urea nitrogen (BUN), serum albumin, creatinine, and C-reactive protein (CRP), all measured upon ICU admission.Vital signs: Mean arterial pressure (MAP) and heart rate. Clinical severity scores: Acute Physiology and Chronic Health Evaluation (APACHE) II and Sequential Organ Failure Assessment (SOFA) scores.To evaluate the prognostic impact of the blood urea nitrogen-to-albumin ratio (BAR), To evaluate the prognostic impact of the blood urea nitrogen-to-albumin ratio (BAR), patients were categorized into four groups based on the quartiles of their baseline log_2_BAR values: Q1 (< 3.58), Q2 (3.58–4.09), Q3 (4.09–4.57), and Q4 (>4.57).

### Exposures

The blood urea nitrogen-to-albumin ratio (BAR) was calculated using the following formula:


$$\begin{array}{l} BAR=\frac{Blood\;Urea\;Nitrogen\left(mg/dL\right)}{SerumÃlbumin\left(g/dL\right)}\left(20\right) \end{array}$$

With the final values expressed in mg/g.

### Outcome

The primary outcome of this study was 28-day all-cause mortality, defined as death from any cause within 28 days following the initiation of continuous renal replacement therapy (CRRT). Survival status was determined by reviewing electronic medical records and hospital discharge summaries. For patients who were discharged before the 28-day follow-up period, survival status was verified through telephonic follow-ups or linked records from the national death registry. Patients who remained alive at the end of the 28-day follow-up period were censored at the time of their last follow-up or hospital discharge.

### Statistical analysis

The participants were divided into four groups based on log_2_BAR quartiles. Continuous variables were reported as mean ± standard deviation (SD) or median (interquartile range, IQR). Frequencies and percentages (*n*, %) were used to characterize categorical variables. Statistical analysis of baseline characteristics involved one-way analysis of variance and the Kruskal–Wallis test for normally and non-normally distributed continuous variables, respectively. The chi-squared test was used for categorical variables.Multiple imputations (five iterations) were used to address the missing covariate values. Supplemental Table 1 provides a detailed account of the missing values.

Multivariable Cox proportional hazard regression models were applied to calculate the hazard ratios (HR) and 95% confidence intervals (95% CIs) for the association between log_2_BAR and 28-day mortality. Model 1 was unadjusted. Model 2 was adjusted for age and sex. Model 3 was fully adjusted for age, sex, hypertension (HTN), phosphate, Charlson Comorbidity Index (CCI), creatinine, C-reactive protein (CRP), and SOFA score To capture the true continuous dose-response relationship and eliminate potential distorting leverage caused by extreme tail outliers, a RCS analysis restricted to the 0–99.7th percentile range was used to explore the potential association between log_2_BAR and 28-day mortality risk after adjusting for the variables in Model 3.

Subgroup analyses were performed using Cox proportional hazards regression models to evaluate the consistency of the association across different strata, with adjustments incorporating the variables from Model 3. Kaplan–Meier curves were used to illustrate variations in survival probability among distinct log_2_BAR groups. The incremental predictive value of adding log_2_BAR to the baseline risk Modelwas assessed using the area under the receiver operating characteristic curve (AUC), the continuous net reclassification improvement (NRI), and the integrated discrimination improvement (IDI).The statistical comparison of the ROC curves and the evaluation of differences in AUC between the prediction models were explicitly performed via the DeLong test. The predictive performance of the models was assessed using calibration curves to evaluate the agreement between predicted and observed risks. Clinical utility was assessed using Decision Curve Analysis (DCA), which quantifies the net benefit of the Modelacross a range of threshold probabilities. All logistic regression models were rigorously evaluated to confirm their adherence to statistical criteria, including posterior predictive checks, binned residuals, influential observations, and collinearity (Supplementary Figure 2).

To evaluate the robustness of the findings, several sensitivity analyses were performed. The association was replicated by converting log_2_BAR into categorical variables using both tertiles and quintiles to ensure consistency across different stratification strategies. E-values were calculated to quantify the minimum strength of association required for potential unmeasured confounders to explain the observed results.

Statistical analyses were performed using R Statistical Software (v4.2.3; R Foundation for Statistical Computing; http://www.R-project.org) and a Free Statistical Analysis Platform (v2.1.1; Beijing Free Clinical Medical Technology Co., Ltd.). A two-tailed test was used to determine statistical significance, with *P* < 0.05 considered significant.

## Results

### Baseline characteristics

A total of 790 patients with sepsis-associated AKI undergoing CRRT were included in this study, with a mean age of 63.6 ± 14.1 years. The cohort comprised 490 (62.0%) males. The baseline characteristics of the study population, stratified by BAR quartiles (Q1–Q4), are summarized in Table 1. Patients in the highest BAR quartile (Q4, BAR > 4.91) were more likely to have a higher prevalence of hypertension (*P* = 0.004) and diabetes mellitus (*P* = 0.024). Notably, clinical severity indicators, including APACHE II score (*P* = 0.032) and SOFA score (*P* < 0.001), were significantly higher in the Q4 group compared with the lower quartiles. Regarding clinical outcomes, patients in the highest quartile (Q4) exhibited a significantly higher incidence of 28-day mortality (70.7%) compared to those in the Q1 (54.8%), Q2 (59.1%), and Q3 (65.5%) groups (*P* = 0.006).

**Table 1:** Baseline characteristics of the study population stratified by ^a^BAR Quartiles.

Variables	Total	Q1 (*n* = 197)	Q2 (*n* = 198)	Q3 (*n* = 197)	Q4 (*n* = 198)	*P* value
	(*N* = 790)	(<3.73)	(3.73–4.29)	(4.29–4.91)	(>4.91)	
Demographics						
Age. Y	63.6 ± 14.1	60.6 ± 15.7	64.8 ± 12.3	65.1 ± 14.2	64.0 ± 13.8	0.005
Male, *n* (%)	490 (62.0)	115 (58.4)	124 (62.6)	115 (58.4)	136 (68.7)	0.112
BMI (kg/m2)	23.6 ± 4.5	24.0 ± 4.0	23.6 ± 4.2	23.6 ± 4.7	23.1 ± 4.9	0.253
Comorbidities						
MI, *n* (%)	72 ( 9.1)	23 (11.7)	14 (7.1)	19 (9.6)	16 (8.1)	0.410
CHF, *n* (%)	144 (18.2)	47 (23.9)	34 (17.2)	35 (17.8)	28 (14.1)	0.086
Cerevascular_dis, *n* (%)	74 ( 9.4)	15 (7.7)	21 (10.6)	13 (6.6)	25 (12.7)	0.151
PVD, *n* (%)	31 ( 3.9)	6 (3)	5 (2.5)	10 (5.1)	10 (5.1)	0.425
Dementia, *n* (%)	27 ( 3.4)	7 (3.6)	4 (2)	6 (3)	10 (5.1)	0.413
DM, *n* (%)	269 (34.1)	50 (25.4)	71 (36)	77 (39.1)	71 (35.9)	0.024
HTN, *n* (%)	418 (52.9)	90 (45.7)	94 (47.5)	114 (57.9)	120 (60.6)	0.004
COPD, *n* (%)	68 ( 8.6)	19 (9.6)	20 (10.1)	14 (7.1)	15 (7.6)	0.645
CCI	3.0 (2.0, 5.0)	2.0 (1.0, 4.0)	3.0 (2.0, 5.0)	3.0 (2.0, 5.0)		0.006
Lab & physiology						
Phosphate (mg/dL)	3.0 (2.0, 5.0)	2.0 (1.0, 4.0)	2.0 (2.0, 4.8)	3.0 (2.0, 5.0)	3.0 (2.0, 5.0)	0.016
BAR	5.6 ± 2.3	5.4 ± 2.2	5.5 ± 2.4	5.7 ± 2.3	6.0 ± 2.2	0.123
MAP (mmHg)	23.2 ± 13.8	9.7 ± 2.3	16.4 ± 1.8	24.7 ± 2.9	42.1 ± 12.7	< 0.001
Hemoglobin (g/dL)	77.5 ± 14.8	76.8 ± 14.4	78.2 ± 15.3	78.2 ± 14.5	76.7 ± 15.0	0.607
BUN (mg/dL)	9.7 ± 2.2	10.1 ± 2.5	10.1 ± 2.4	9.5 ± 1.7	9.2 ± 1.8	< 0.001
Creatinine (mg/dL)	56.9 ± 29.1	28.1 ± 8.8	43.0 ± 9.1	63.4 ± 14.0	93.0 ± 26.0	< 0.001
Albumin (g/dL)	2.7 ± 1.5	1.9 ± 0.9	2.5 ± 1.2	2.9 ± 1.7	3.3 ± 1.6	< 0.001
CRP (mg/L)	2.6 ± 0.6	2.9 ± 0.6	2.6 ± 0.5	2.6 ± 0.5	2.3 ± 0.5	< 0.001
Severity & treatment						
MV_CRRT, *n* (%)	625 (79.2)	163 (82.7)	161 (81.3)	149 (76)	152 (76.8)	0.267
APACHE II score	27.4 ± 8.0	26.1 ± 8.0	27.4 ± 7.7	27.8 ± 8.2	28.3 ± 7.9	0.032
SOFA score	12.0 ± 3.5	11.2 ± 3.5	12.1 ± 3.3	12.1 ± 3.6	12.6 ± 3.7	< 0.001
Outcomes						
^b^STATUS 28, *n* (%)	494 (62.5)	108 (54.8)	117 (59.1)	129 (65.5)	140 (70.7)	0.006
S28TIME (days)	18.3 ± 11.6	19.6 ± 11.4	20.2 ± 11.1	17.5 ± 11.7	16.2 ± 11.9	0.002

APACHE II, Acute Physiology and Chronic Health Evaluation II; BAR, blood urea nitrogen-to-albumin ratio(mg/g); BMI, body mass index; BUN, blood urea nitrogen; CCI, Charlson comorbidity index; Cerevascular_dis, cerebrovascular disease; CHF, congestive heart failure; COPD, chronic obstructive pulmonary disease; CRP, C-reactive protein; DM, diabetes mellitus; HTN, hypertension; MAP, mean arterial pressure; MI, myocardial infarction; MV_CRRT, mechanical ventilation or continuous renal replacement therapy; PVD, peripheral vascular disease; S28TIME, time to 28-day outcome; SOFA, Sequential Organ Failure Assessment; STATUS 28, 28-day mortality. Data are presented as mean ± SD for normally distributed continuous variables, median (IQR) for skewed continuous variables, or number (percentage) for categorical variables. One-way ANOVA for normally distributed continuous variables; Kruskal-Wallis test for skewed continuous variables; Chi-squared test or Fisher's exact test for categorical variables. ^a^Categorized into 4 levels based on BAR Quartiles: Q1 (< 3.73), Q2 (3.73–4.29), Q3 (4.29–4.91),and Q4(>4.91). ^b^STATUS 28 was defined as all-cause mortality within 28 days of ICU admission.

### Association between BAR and 28-day mortality

Multivariate adjustment revealed a significant association between log_2_BAR and 28-day mortality (Table 2). An increment of one unit in log_2_BAR corresponded to a 33% greater risk of 28-day mortality (HR = 1.33, 95% CI: 1.15–1.53, *P* < 0.001). Individuals in the highest log_2_BAR quartile exhibited a 75% higher risk of 28-day mortality than those in the lowest log_2_BAR quartile (adjusted HR = 1.75, 95% CI: 1.26–2.43, *P* = 0.001). Supplementary Figure 3 illustrates the multivariate-adjusted RCS analysis examining the link between the log_2_BAR and mortality risk. A positive linear relationship was observed (*P* for nonlinearity = 0.425).

**Table 2:** Multivariable cox regression analysis of the association between log_2_(BAR) Quartiles and 28-day mortality.

Categories	Model 1		Model 2		Model 3	
	HR (95%CI)	*P* value	HR (95%CI)	*P* value	HR (95%CI)	*P* value
log_2_(BAR)	1.23 (1.10~1.37)	< 0.001	1.23 (1.10~1.37)	< 0.001	1.33 (1.15~1.53)	< 0.001
log_2_(BAR) quartiles						
Q1 (< 3.73)	1 (Ref)		1 (Ref)		1 (Ref)	
Q2 (3.73–4.29)	1.06 (0.82~1.38)	0.662	1.05 (0.81~1.37)	0.714	0.92 (0.67~1.26)	0.597
Q3 (4.29–4.91)	1.33 (1.03~1.72)	0.029	1.31 (1.01~1.7)	0.04	1.44 (1.05~1.98)	0.024
Q4 (>4.91)	1.55 (1.21~1.99)	0.001	1.54 (1.20~1.99)	0.001	1.75 (1.26~2.43)	0.001
*P* for trend		< 0.001		< 0.001		< 0.001

AKI, acute kidney injury; BAR, blood urea nitrogen-to-albumin ratio (mg/g); CI, confidence interval; HR, hazard ratio; Ref, reference group. Cox proportional hazards models were used to estimate hazard ratios (HRs) and 95% confidence intervals (CIs) for 28-day mortality. log_2_(BAR) was calculated as the base-2 logarithm of the BAR to normalize the data, and HRs represent the risk per doubling of the ratio. Model 1:Non-adjusted. Model 2: adjusted for age, Sex. Model 3:adjusted for age, Sex, HTN, Phosphate, CCI, creatinine, CRP, SOFA score.

### Subgroup analysis and Kaplan–Meier curves

**Figure 1:**
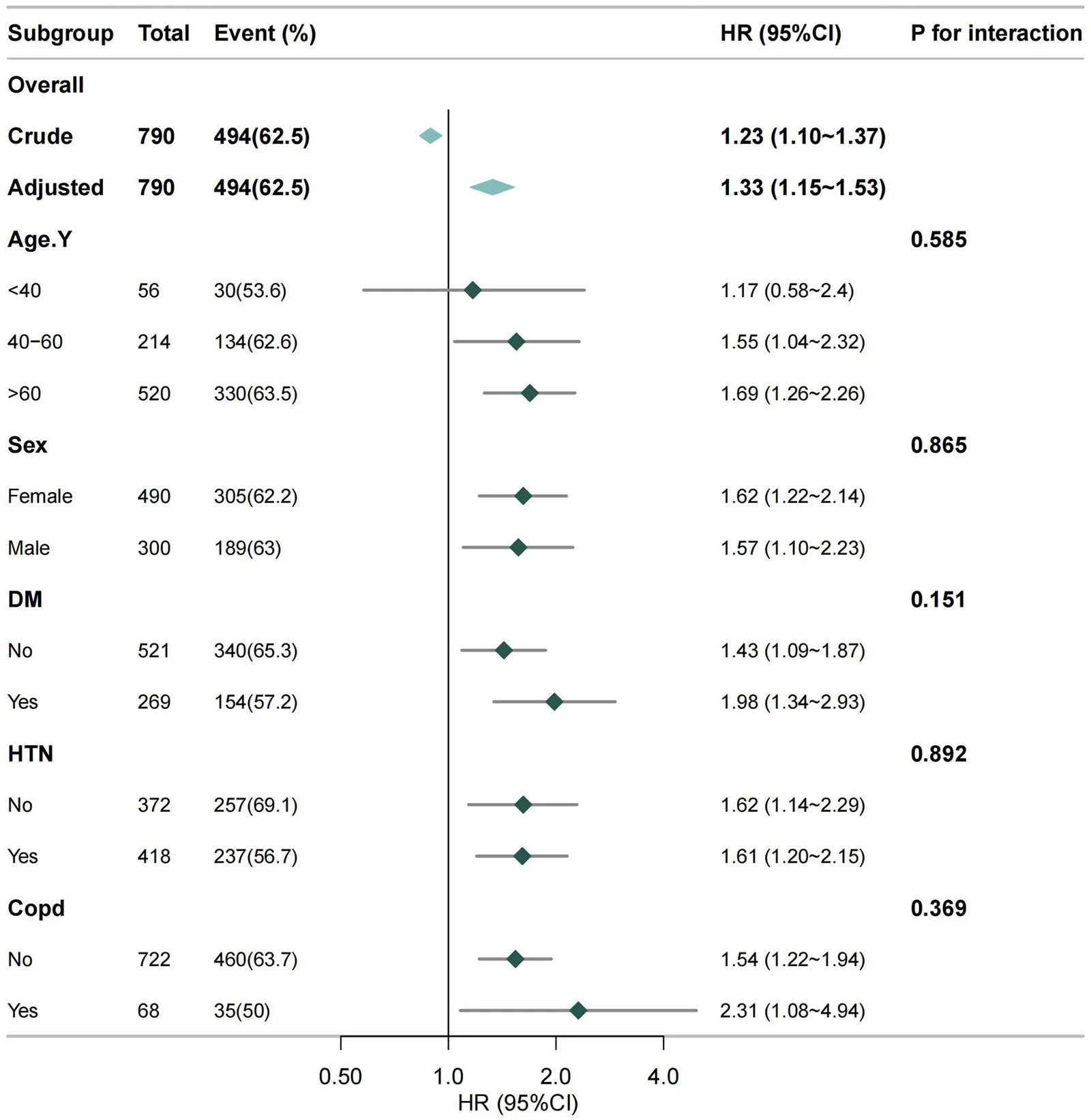
Subgroup Analysis of BAR and 28-day mortality risk using multivariable cox regression model. Forest plot displaying the adjusted hazard ratios (HRs) and 95% confidence intervals (CIs) for 28-day mortality across various subgroups. BAR: blood urea nitrogen-to-albumin ratio (mg/g), HR, hazard ratio; CI, confidence interval; DM, diabetes mellitus; HTN, hypertension. The analysis was conducted using a multivariable Cox regression model, adjusting for age, sex, BMI, comorbidities (MI, CHF, Cerevascular_dis, PVD, Dementia, DM, HTN, COPD), CCI, MAP, Hemoglobin, and SOFA score. The overall crude and adjusted models included 790 patients. The diamond represents the overall effect size, and the horizontal lines indicate the 95% CIs. *P*-values for interaction were calculated to assess the consistency of the association between BAR and 28-day mortality across different subgroups (*P* for interaction > 0.05 for all).

**Figure 2:**
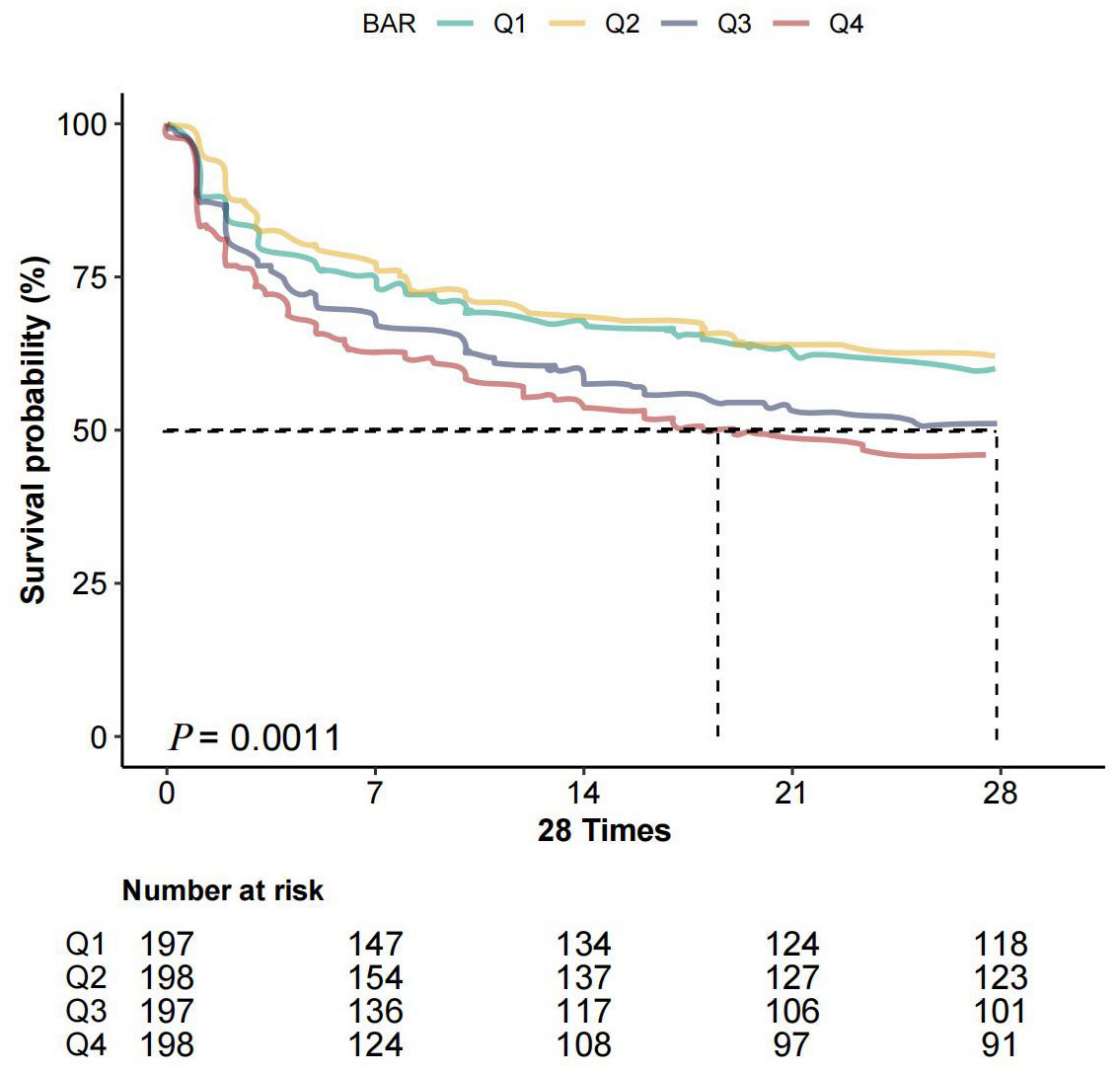
Kaplan–Meier survival curves for 28-day mortality across log_2_BAR Quartiles. The curves illustrate the cumulative survival probability over the 28-day follow-up period for four groups categorized by BAR quartiles (Q1, Q2, Q3, and Q4). The number of patients at risk for each group is provided at 0, 7, 14, 21, and 28 days. The log-rank test for intergroup comparison yields a *P*-value of 0.0011, indicating a statistically significant difference in 28-day survival probability across the four BAR quartiles. Patients in the highest BAR quartile (Q4) exhibit the lowest survival probability throughout the follow-up duration compared to those in the Q1, Q2, and Q3 groups.

Subgroup analyses were conducted to evaluate the consistency of the association between log_2_BAR and 28-day mortality across various clinical and demographic categories (Figure 1). As shown in the forest plot, the association remained consistent across subgroups, including age, sex, diabetes mellitus (DM), hypertension (HTN), and chronic obstructive pulmonary disease (COPD). No significant interactions were observed across these strata (*P* for interaction > 0.05 for all).The cumulative survival probability was further analyzed using Kaplan-Meier curves stratified by log_2_BAR quartiles (Figure 2). A significant separation in survival trajectories was observed among the four groups (Log-rank test, *P* = 0.0011). Notably, patients in the highest log_2_BAR quartile (Q4) exhibited a significantly lower survival probability compared to those in the Q1, Q2, and Q3 groups throughout the 28-day follow-up period. The number of patients at risk, provided at 0, 7, 14, 21, and 28 days, confirms the progressive divergence in mortality risk among the quartiles.

### Incremental predictive value of log_2_BAR

**Figure 3:**
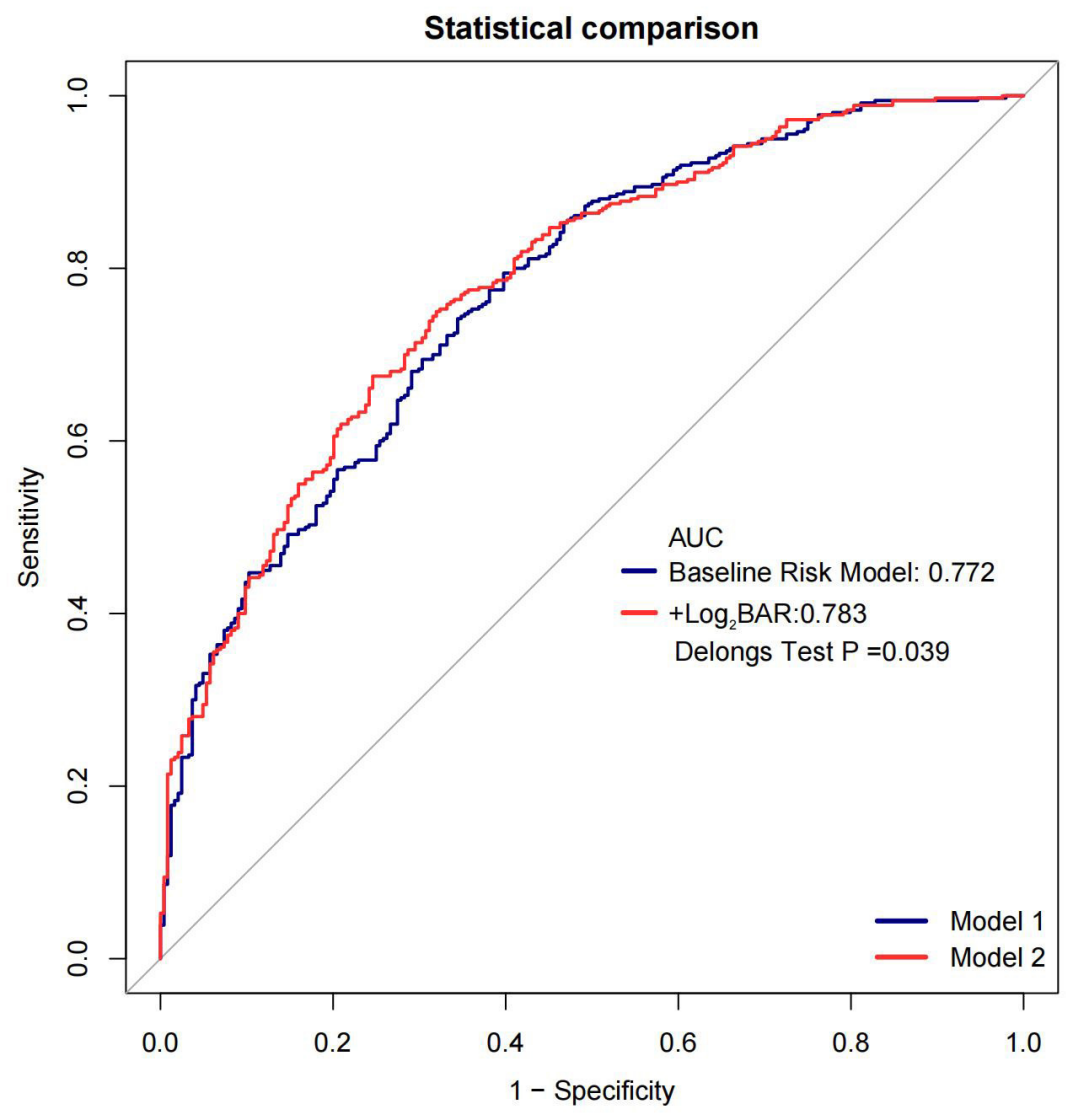
Comparison of ROC curves for 28-day mortality prediction models. The receiver operating Characteristic (ROC) curves illustrate and compare the predictive performance of two models for 28-day mortality in patients with sepsis-associated AKI. Model 1 (blue line) demonstrates an area under the curve (AUC) of 0.772 (95% CI: 0.735–0.809). Model 2 (red line), which incorporates the blood urea nitrogen-to-albumin ratio (BAR) (mg/g), shows an AUC of 0.783 (95% CI: 0.747–0.820). The statistical comparison between the two models yielded a *P* = 0.039, indicating no statistically significant difference in predictive discrimination.

The incremental predictive value of adding log_2_BAR to the baseline risk Model was assessed using ROC, NRI, and IDI (Figure 3, Table 3). The AUC increased from 0.772 in the baseline Model to 0.783 after incorporating log_2_BAR. Notably, the statistical significance of this absolute AUC increment was formally confirmed by the DeLong test (*P* = 0.0039). Additionally, individual ROC curve analyses of standalone parameters showed that the standalone SOFA score (AUC = 0.697) and standalone APACHE II score (AUC = 0.599) exhibited higher discriminative trajectories than the standalone log_2_BAR index (AUC =0.579) when evaluated in isolation (Supplementary Figure 6). Furthermore, the addition of log_2_BAR resulted in a significant improvement in risk reclassification, with a continuous NRI of 0.3736 (95% CI: 0.2198–0.5274, *P* < 0.001) and an IDI of 0.0167 (95% CI: 0.006–0.0275, *P* = 0.002), To further evaluate the prognostic performance and clinical applicability of log_2_BAR, we performed calibration curve analysis and decision curve analysis (DCA).Calibration: As shown in Supplementary Figure 5A, the Model incorporating log_2_BAR (blue line) demonstrated valuable calibration compared to the baseline risk Model (red line), tracking more closely to the ideal reference line for 28-day mortality prediction.Clinical Net Benefit: As presented in Supplementary Figure 5B, DCA revealed that the Modelincorporating log_2_BAR consistently provided a higher net benefit than the baseline model, the “treat-all” strategy, and the “treat-none” strategy across a wide range of threshold probabilities.

**Table 3:** Incremental predictive value of adding log_2_BAR to the baseline risk Modelfor 28-day mortality prediction.

Model	NRI (95%Cl)	*P*	IDI (95%Cl)	*P*
Baseline risk model	Ref.	Ref.	Ref.	Ref.
+log_2_BAR	0.3736 [0.2198–0.5274]	< 0.001	0.0167 [0.006–0.0275]	0.002

The baseline risk modelincludes age, sex, BMI, comorbidities, CCI, MAP, hemoglobin, and SOFA score. log_2_BAR, log2-transformed blood urea nitrogen-to-albumin ratio. Data are presented as point estimates with 95% confidence intervals unless otherwise indicated.

### Sensitivity analysis

Multivariable Cox regression analysis was performed to evaluate the stability of the association between log_2_BAR and 28-day mortality (Supplementary Tables 2, 3). A one-unit increase inlog_2_BAR was consistently associated with an increased hazard of 28-day mortality across all models. When categorized into quintiles, individuals in the highest log_2_BAR quintile (Q5) demonstrated a 77% greater risk of 28-day mortality than those in the lowest quintile (Q1) (HR = 1.77, 95% CI: 1.24–2.52, *P* = 0.002). Moreover, the findings remained robust when different categorical stratification strategies (tertiles vs. quintiles) were employed. Finally, E-values were computed to evaluate the influence of potential unmeasured confounders. The association between log_2_BAR and the risk of 28-day mortality was robust unless unmeasured confounding factors were associated with both the log_2_BAR and the risk of mortality at a relative risk exceeding 1.73 (Supplementary Figure 4).

## Discussion

This prospective cohort study of 790 patients with sepsis-associated AKI treated with CRRT reveals several noteworthy findings. First, an elevated log_2_BAR was independently correlated with a higher risk of 28-day mortality in patients with sepsis-associated AKI undergoing CRRT. This association remained significant after comprehensive adjustment for baseline severity scores, comorbidities, and clinical laboratory parameters. Second, the dose-response relationship between log_2_BAR and mortality was found to be monotonic and linear, with no evidence of a threshold effect. Furthermore, sensitivity analyses, including categorical stratification and the calculation of E-values, confirmed that this association was robust and unlikely to be fully explained by potential unmeasured confounders. These findings imply that the BAR serves as a dynamic and integrated indicator of protein catabolism and systemic inflammation, providing supplementary prognostic insights alongside conventional clinical evaluation metrics.Previous research on prognostic biomarkers for sepsis-associated AKI has left several critical issues unresolved (19–21). First, while existing studies have established the prognostic value of isolated markers like serum creatinine or albumin, they have not yet evaluated the integrated metabolic stress quantified by BAR—a metric that captures the critical balance between renal nitrogen excretion and systemic nutritional status—despite its proven utility in other critical care contexts (22–24). Second, methodological limitations such as reliance on small-scale single-center designs or insufficient adjustment for time-varying confounders have constrained prognostic inference (25–27). For example, a study conducted in 2020 identified associations between albumin levels and mortality in AKI (28); however, the potential synergistic effect of BUN was not investigated. Similarly, a study conducted in 2023 failed to consider the intensive management factors during CRRT, which may influence both BUN and albumin levels (22). This study contributes to the advancement of this field through three key innovations: (1) By using a prospective clinical cohort, we rigorously identified 28-day outcomes, ensuring sufficient statistical power to detect robust associations. (2) The use of restricted cubic spline (RCS) analysis characterized the continuous dose-response relationship between log_2_BAR and mortality, providing prognostic insights previously not reported in CRRT populations, In our study, after excluding extreme tail outliers (restricting to the 0–99.7th percentile range), the RCS analysis demonstrated a strictly monotonic, linear association between log_2_BAR and 28-day mortality *P* for non-linearity = 0.425,*P*_overall_ < 0.001).

While recent literature on general sepsis populations has identified an inflection point for BAR (approximately 20.91 mg/g), our threshold effect analysis did not reveal a similar saturation point in this CRRT-treated cohort. This divergence likely stems from the metabolic impact of continuous renal replacement therapy. The standardized effluent dose (≥35 ml/kg/h) utilized in our patients provides substantial extrinsic clearance of BUN, which may mitigate the extreme azotemic surges and linearize the prognostic impact of the BAR index compared to patients managed conservatively.While the absolute geometric increment in AUC after adding log_2_BAR was relatively modest (AUC = 0.011), this improvement was formally confirmed to be highly statistically significant by the DeLong test (*P* = 0.0039). Furthermore, the addition of this marker yielded prominent improvements in risk reclassification indices, as demonstrated by the Net Reclassification Improvement (continuous NRI = 0.3736, *P* < 0.001) and Integrated Discrimination Improvement (IDI = 0.0167, *P* = 0.002). We acknowledge that when evaluated in complete isolation, the standalone log_2_BAR index exhibits a lower discriminative trajectory compared to established, multi-parametric severity scores like the standalone SOFA and APACHE II scores (Supplementary Figure 6). This hierarchical divergence is pathologically expected, as the clinical definition and diagnostic criteria of sepsis inherently rely on the multi-organ clinical variables aggregated within the SOFA score. A single laboratory ratio is not intended to substitute for such extensive macro-clinical clinical staging systems. Crucially, however, the primary prognostic merit of log_2_BAR lies in its incremental and complementary utility rather than its performance as an isolated tool. While macro-clinical scores evaluate visible organ dysfunction, log_2_BAR specifically captures the underlying severe metabolic-catabolic stress and acute protein degradation. Even though our comprehensive baseline model already stringently incorporated the complete SOFA score as a core covariate, the further inclusion of log_2_BAR successfully yielded an independent, statistically significant upward shift in overall discriminative capacity (*P* = 0.0039).

In critically ill settings, even a small increase in discriminative power, when paired with high net reclassification improvement, can lead to more accurate identification of high-risk patients who might otherwise be misclassified by traditional scores alone. Furthermore, the model incorporating log_2_BAR demonstrated good calibration and significant clinical net benefit, as evidenced by decision curve analysis (DCA), confirming its utility for early prognostic stratification in high-risk patients. (3) Rigorous Bias Mitigation and Robustness Testing: We implemented a multi-layered approach to address statistical uncertainties and minimize potential biases. This included multiple imputation to handle missing covariates, categorical sensitivity analyses (using tertiles and quintiles) to validate findings across different stratification strategies, and E-value quantification to determine the minimum strength of association required for any unmeasured confounder to negate our observed results. These measures collectively confirm the stability and clinical reliability of log_2_BAR as an independent prognostic marker. The association between log_2_BAR and 28-day mortality is hypothesized to be linked with. interconnected pathways encompassing hypercatabolism and systemic inflammatory response (29). Elevated BAR is continuous with clinically inferred protein degradation

and impaired nutritional state, which are hallmark metabolic signatures of the sepsis-associated inflammatory storm (30). This metabolic shift impairs mitochondrial function and reduces the synthesis of essential transport proteins, thereby exacerbating organ failure (31, 32). Concurrently, severe AKI leads to profound nitrogen retention, which, when coupled with systemic hypoalbuminemia, reflects a state of advanced metabolic imbalance (33, 34). These metabolic abnormalities play significant roles in multi-organ dysfunction syndrome through various mechanisms. Hypercatabolism exacerbates the inflammatory state, establishing a detrimental cycle that perpetuates systemic instability (35). The biological significance of BAR in the context of CRRT warrants deeper exploration. Unlike general ICU populations, patients in our cohort were subject to continuous extrinsic urea clearance. In such settings, an elevated BAR signifies a profound metabolic-clearance mismatch. A high BUN level despite standardized CRRT (≥35 ml/kg/h) reflects aggressive protein catabolism and nitrogenous waste production that overwhelms the dialytic capacity, marking a state of conceptually inferred “refractory hypercatabolism” driven by the underlying septic storm. Simultaneously, the albumin component in these patients faces a “double-hit”—attrition from systemic inflammatory capillary leakage and potential protein loss across the CRRT membrane. Consequently, the log_2_BAR index serves as a surrogate barometer of the balance between catabolic stress and nutritional-immunological reserve.The practical utility of the log_2_BAR index lies in its ability to bridge laboratory data to bedside decision-making. For clinicians managing sepsis-associated AKI undergoing CRRT, a log_2_BAR value in the highest quartile (>4.57 mg/g, Q4) could serve as a potential conceptual “metabolic red flag” to prompt closer evaluation. Specifically, high-BAR patients may benefit from enhanced nutritional surveillance and tailored protein supplementation to counteract the severe hypercatabolism reflected by this ratio. Furthermore, a persistently high or rising BAR during treatment suggests a potential metabolic-clearance mismatch, implying that the standard CRRT prescription may be insufficient to keep pace with aggressive endogenous nitrogenous waste production. This clinical signal could potentially warrant a closer evaluation of dialytic adequacy, such as inspecting membrane patency or considering a cautious upward titration of the effluent dose to maintain optimal clearance. Nonetheless, we emphasize that these recommendations represent a conceptual paradigm for personalized risk management. Further prospective interventional studies and randomized controlled trials (RCTs) are strictly required to confirm whether BAR-guided therapeutic adjustments can formally translate into a tangible survival benefit.

**Figure 4:**
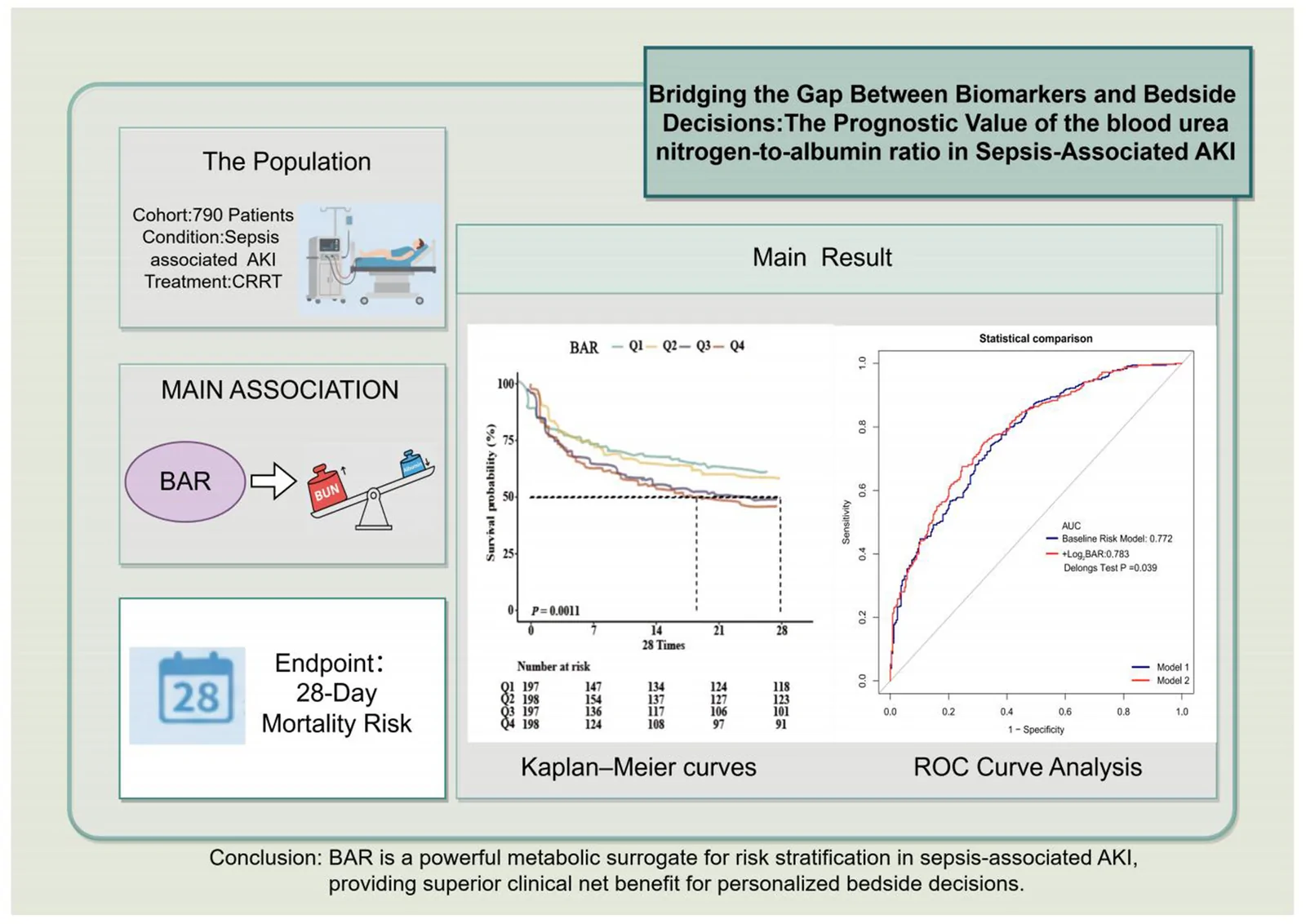
Graphical summary of the study design and main findings. The study included 790 patients with sepsis-associated acute kidney injury undergoing continuous renal replacement therapy. Higher blood urea nitrogen-to-albumin ratio (BAR) values were associated with higher 28-day mortality and Kaplan-Meier and receiver operating characteristic analyses were used to evaluate prognostic performance. AKI, acute kidney injury; BAR, blood urea nitrogen-to-albumin ratio; CRRT, continuous renal replacement therapy; ROC, receiver operating characteristic.

Consequently, the BAR may serve as a valuable complementary conceptual indicator indicator of acute metabolic status in critically ill patients, capturing the interaction between acute inflammatory stress and nutritional depletion that collectively drives poor clinical outcomes. A graphical summary of the study design and principal findings is presented in Figure 4.

### Strengths and limitations

Our study possesses several key strengths that significantly advance prognostic strategies for patients with sepsis-associated acute kidney injury (AKI) undergoing continuous renal replacement therapy (CRRT). (1) The BAR, an easily accessible metric derived from routine blood chemistry, was identified as a robust and independent predictor of 28-day mortality. This enables cost-effective and timely risk stratification in intensive care settings without the need for specialized or expensive biomarkers. (2) By employing RCS analysis and incremental predictive metrics (NRI, IDI), we demonstrated that BAR provides valuable incremental prognostic insights compared to traditional clinical severity scores alone, highlighting it as a dynamic, “value-added” risk-stratification tool. (3) Focusing on the CRRT-treated cohort addresses a critical clinical gap, as patients in this category exhibit profound metabolic derangements—such as hypercatabolism and systemic inflammation—that are often inadequately captured by static scoring systems. By leveraging a prospective cohort and rigorous statistical methods (multiple imputation, E-value analysis, and subgroup validation), the results advocate for the integration of BAR into clinical monitoring protocols to improve patient management in the ICU.

This study had some limitations. First, the cohort exclusively comprised patients with sepsis-associated AKI treated with CRRT in a single clinical system in Korea, representing a highly selected subgroup of critically ill patients with severe sepsis-associated AKI and profound metabolic failure. This profound population specificity introduces a notable selection bias, which inherently limits the direct generalizability and extrapolation of our findings to alternative geographic populations (such as Western cohorts), patients with milder or alternative AKI etiologies, or general septic patients who do not require invasive renal replacement therapy, where the baseline distribution, prognostic performance, and clinical utility of the BAR index may differ substantially or potentially diminish.; however, the strict implementation of standardized management protocols helps enhance the internal validity of these specific results. Second, despite comprehensive multivariable adjustments and E-value analyses (minimum E-value: 1.73), residual confounding from unmeasured variables—such as specific nutritional intervention protocols, varying CRRT modalities, and the administration of exogenous albumin during the study period—cannot be entirely excluded as these data were not available in the primary registry. Crucially, due to the nature of the primary registry database, direct assessments regarding patient baseline metabolic rates (e.g., indirect calorimetry), dynamic cytokine profiles, or quantitative body composition (such as muscle-to-fat ratio or lean body mass) were not available, meaning our inferred mechanistic pathways should be interpreted cautiously as plausible biological hypotheses rather than definitive measures." Nevertheless, the E-value suggests that unmeasured confounders would need risk ratios to negate the findings, which is statistically unlikely given the known biological effect sizes of nitrogen and albumin metabolic shifts in critical illness. Third, while missing data were addressed using multiple imputation, selection bias may persist due to the observational nature of the registry. Sensitivity analyses conducted across the imputed and complete-case datasets yielded consistent results, supporting the robustness of our findings. This study relied on a single baseline BAR measurement. While our results confirm its early prognostic value, we acknowledge that serial monitoring of BAR trajectories might provide valuable real-time risk assessment. Future multicenter cohort studies that incorporate serial monitoring of metabolic markers and inflammatory cytokines can further validate these findings. Despite these limitations, this study provides novel and methodologically rigorous evidence that BAR is a potent prognostic indicator in critically ill patients, thereby contributing to the development of targeted, patient-specific management strategies.

## Conclusion

This prospective cohort study robustly established the log_2_BAR as a clinically accessible and independent prognostic indicator for 28-day mortality in patients with sepsis-associated AKI undergoing CRRT. By capturing the dynamic interplay between systemic protein hypercatabolism and nutritional depletion, log_2_BAR provides significant incremental predictive value beyond traditional severity scoring systems, offering a more nuanced understanding of metabolic stress in critically ill patients.These findings may complement patient care by supporting the integration of BAR into routine clinical monitoring protocols and highlighting the potential utility of targeted metabolic management—such as optimized nutritional support and inflammation management—to improve clinical outcomes in the ICU. By refining early risk stratification and providing an actionable pathway for personalized management, the BAR serves as a potent, non-invasive tool to mitigate the high mortality burden in patients with sepsis-associated AKI.

## Data Availability

The original contributions presented in the study are included in the article/supplementary material, further inquiries can be directed to the corresponding author.
