# Supplementary material for: Bridging the gap between biomarkers and bedside decisions: the prognostic value of the blood urea nitrogen-to-albumin ratio in sepsis-associated AKI
Source: Front Nutr. 2026 Aug 13;13:1831995. doi: 10.3389/fnut.2026.1831995 (PMC13518199; doi:10.3389/fnut.2026.1831995)
Supplement: Supplementary file 1 [file Image_1.pdf]

Supplementary Fig 1 Flowchart of Patient Selection

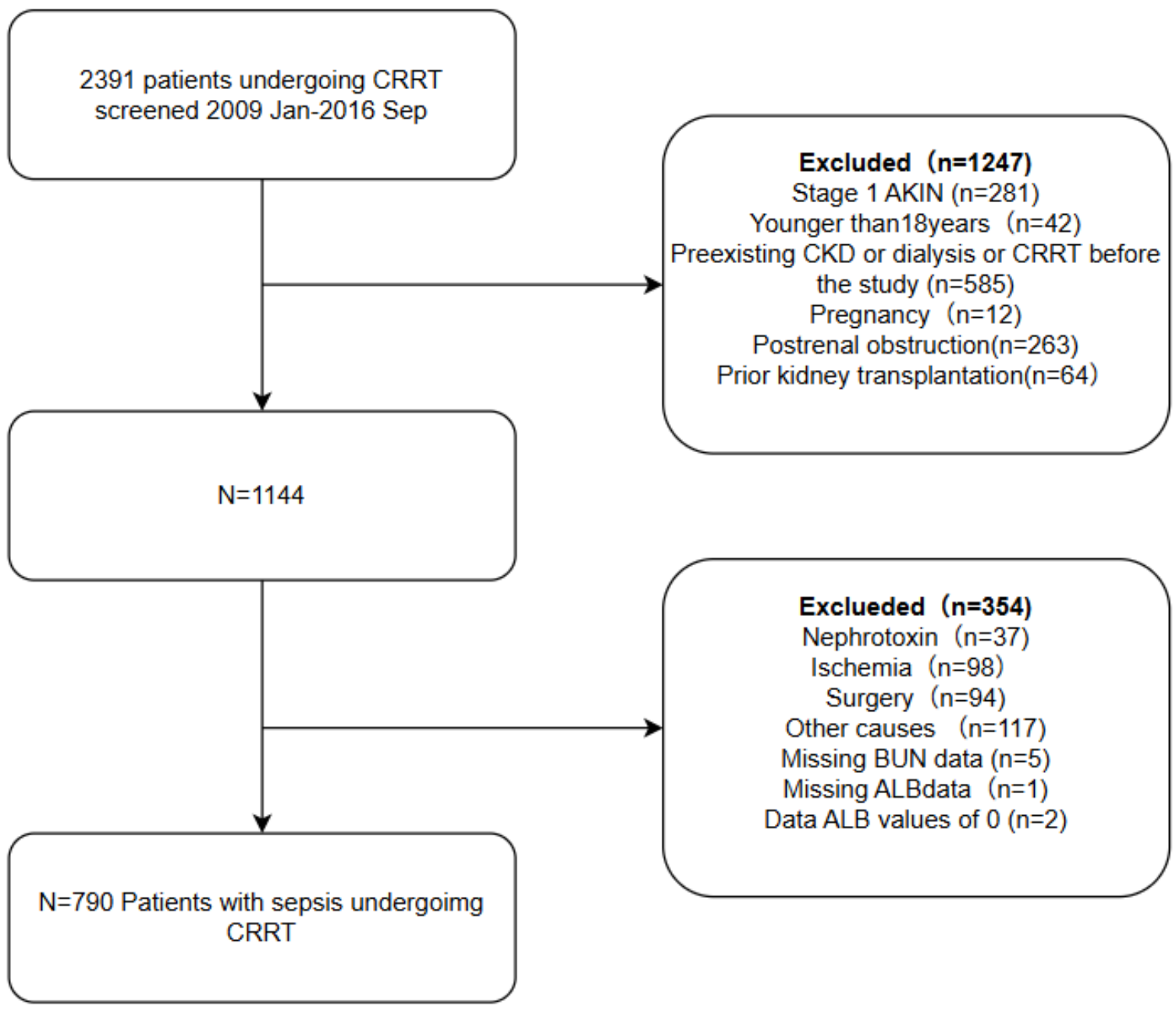

Abbreviations: AKI, acute kidney injury; AKIN, Acute Kidney Injury Network; ALB, albumin; APACHE II, Acute Physiology and Chronic Health Evaluation II; BAR, blood urea nitrogen-to-albumin ratio; BUN, blood urea nitrogen; CKD, chronic kidney disease; CRP, C-reactive protein; CRRT, continuous renal replacement therapy; CVVHDF, continuous venovenous haemodiafiltration; ICU, intensive care unit; MAP, mean arterial pressure; SOFA, Sequential Organ Failure Assessment.
