# Supplementary material for: Bridging the gap between biomarkers and bedside decisions: the prognostic value of the blood urea nitrogen-to-albumin ratio in sepsis-associated AKI
Source: Front Nutr. 2026 Aug 13;13:1831995. doi: 10.3389/fnut.2026.1831995 (PMC13518199; doi:10.3389/fnut.2026.1831995)
Supplement: Supplementary file 2 [file Image_2.pdf]

Supplementary Figure 2. Diagnostic plots for the multivariate logistic regression model.

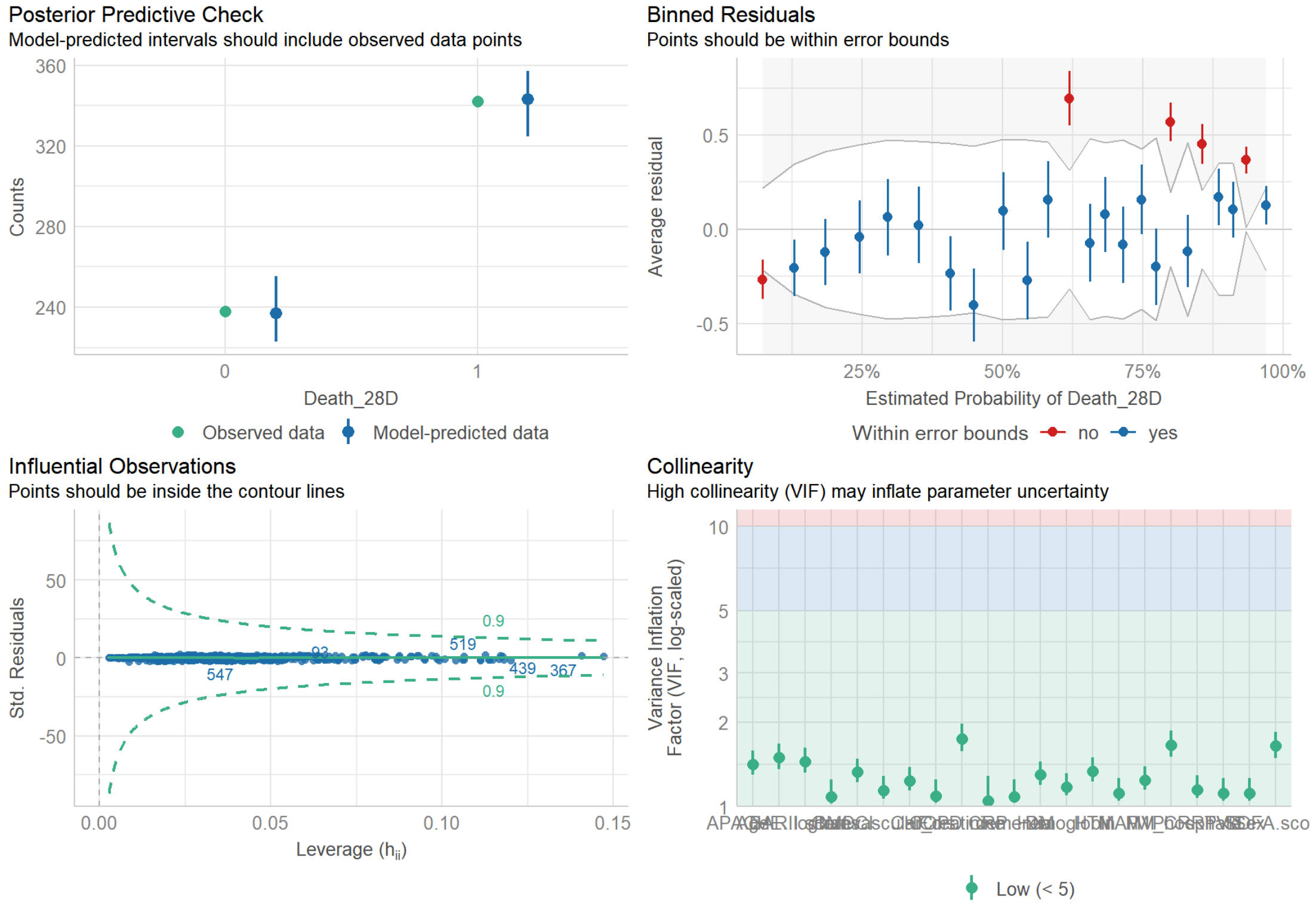


This figure displays a comprehensive assessment of the model fit and statistical assumptions for the logistic regression analysis.

(A) Posterior Predictive Check (Top Left): The plot compares the observed data (green dots) with the model-predicted intervals (blue lines). The close alignment between the observed and predicted distributions suggests that the model accurately captures the underlying data structure.

(B) Binned Residuals (Top Right): This plot evaluates the agreement between observed and predicted probabilities across binned risk groups. Although minor deviations are observed, the majority of points fall within the simulated error bounds (grey area), indicating an overall adequate model fit.

(C) Influential Observations (Bottom Left): The plot of standardized residuals against leverage identifies potential outliers. All observations fall within the Cook's distance contour lines, confirming that no single influential data point is disproportionately driving the model estimates.

(D) Collinearity (Bottom Right): The Variance Inflation Factor (VIF) plot assesses multicollinearity among the included covariates, including age, Crea, SOFA score, and $\log_2$BAR. All VIF values are well below the threshold of 5 (green dots), indicating that multicollinearity is not a confounding factor in the model.
