# Supplementary material for: Bridging the gap between biomarkers and bedside decisions: the prognostic value of the blood urea nitrogen-to-albumin ratio in sepsis-associated AKI
Source: Front Nutr. 2026 Aug 13;13:1831995. doi: 10.3389/fnut.2026.1831995 (PMC13518199; doi:10.3389/fnut.2026.1831995)
Supplement: Supplementary file 3 [file Image_3.pdf]

Supplementary Fig 3. The dose-response relationship between $\log_2$BAR and the risk of 28-day mortality.

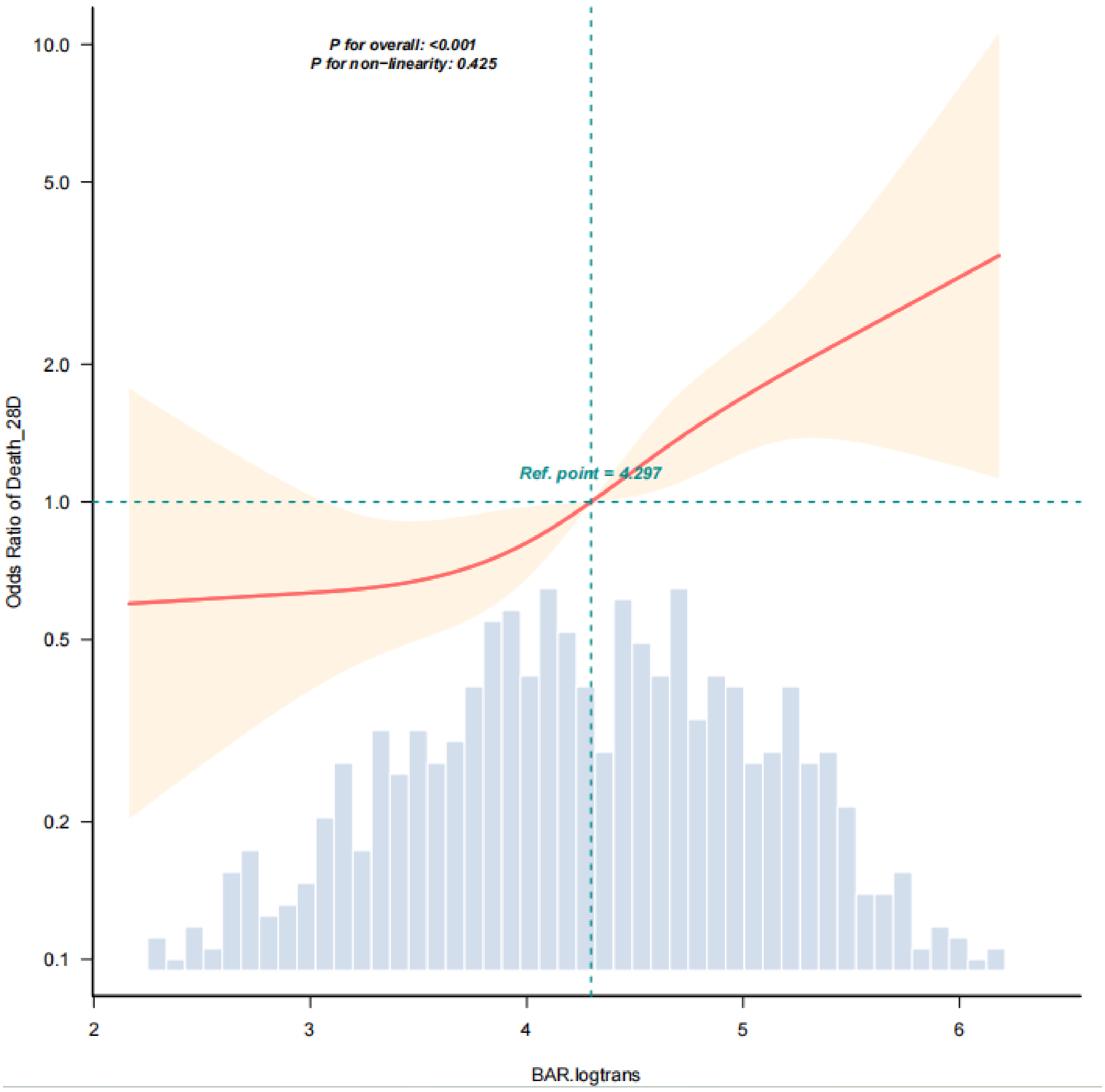

Log_2BAR: logarithmically transformed blood urea nitrogen-to-albumin ratio (base 2). This hazard ratio plot, constructed with restricted cubic splines, depicts the continuous dose-response association between Log_2BAR and the risk of 28-day mortality. To mitigate the potential distorting leverage caused by extreme tail outliers, the analysis is restricted to the 0–99.7th percentile range. The reference point is set at a Log_2BAR value of 4.255. The solid red line indicates the estimated hazard ratio, with the red shaded area representing the 95% confidence interval. The dashed line at a hazard ratio of 1.0 serves as the reference for no effect. The overall association is highly statistically significant ($P_{\text{overall}} < 0.001$), while the P-value for non-linearity is 0.425 (P for non-linearity = 0.425), confirming a strictly linear and monotonic relationship.

The analysis is adjusted for covariates as per Model 3 (including age, sex, BMI, comorbidities, CCI, MAP, Hemoglobin, and SOFA score).
