# Supplementary material for: Bridging the gap between biomarkers and bedside decisions: the prognostic value of the blood urea nitrogen-to-albumin ratio in sepsis-associated AKI
Source: Front Nutr. 2026 Aug 13;13:1831995. doi: 10.3389/fnut.2026.1831995 (PMC13518199; doi:10.3389/fnut.2026.1831995)
Supplement: Supplementary file 4 [file Image_4.pdf]

Supplementary Figure 4. Performance evaluation of the prognostic model incorporating log_2BAR.

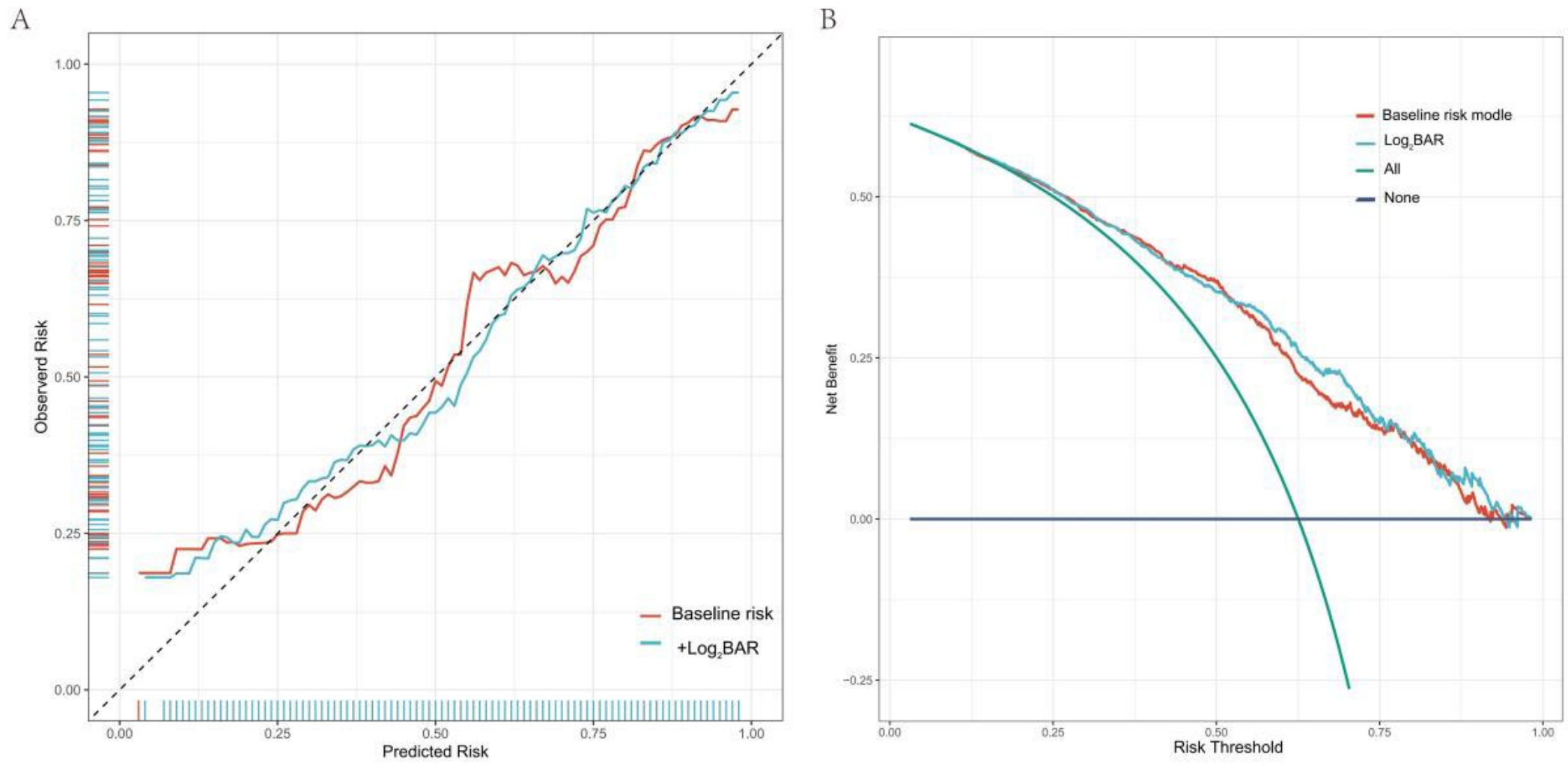

(A) Calibration curve showing the agreement between predicted and observed risks for 28-day mortality. The blue line represents the model including log_2BAR, which demonstrates better calibration compared to the baseline risk model (red line). (B) Decision curve analysis (DCA) demonstrating the clinical net benefit of the prognostic models. The model incorporating log_2BAR (blue line) exhibits a higher net benefit than the baseline model (red line), the "treat-all" strategy (gray dashed line), and the "treat-none" strategy (black solid line) across a wide range of threshold probabilities.
