# Supplementary material for: Bridging the gap between biomarkers and bedside decisions: the prognostic value of the blood urea nitrogen-to-albumin ratio in sepsis-associated AKI
Source: Front Nutr. 2026 Aug 13;13:1831995. doi: 10.3389/fnut.2026.1831995 (PMC13518199; doi:10.3389/fnut.2026.1831995)
Supplement: Supplementary file 5 [file Image_5.pdf]

Supplementary Figure 5.E-value analysis of unmeasured confounding factors

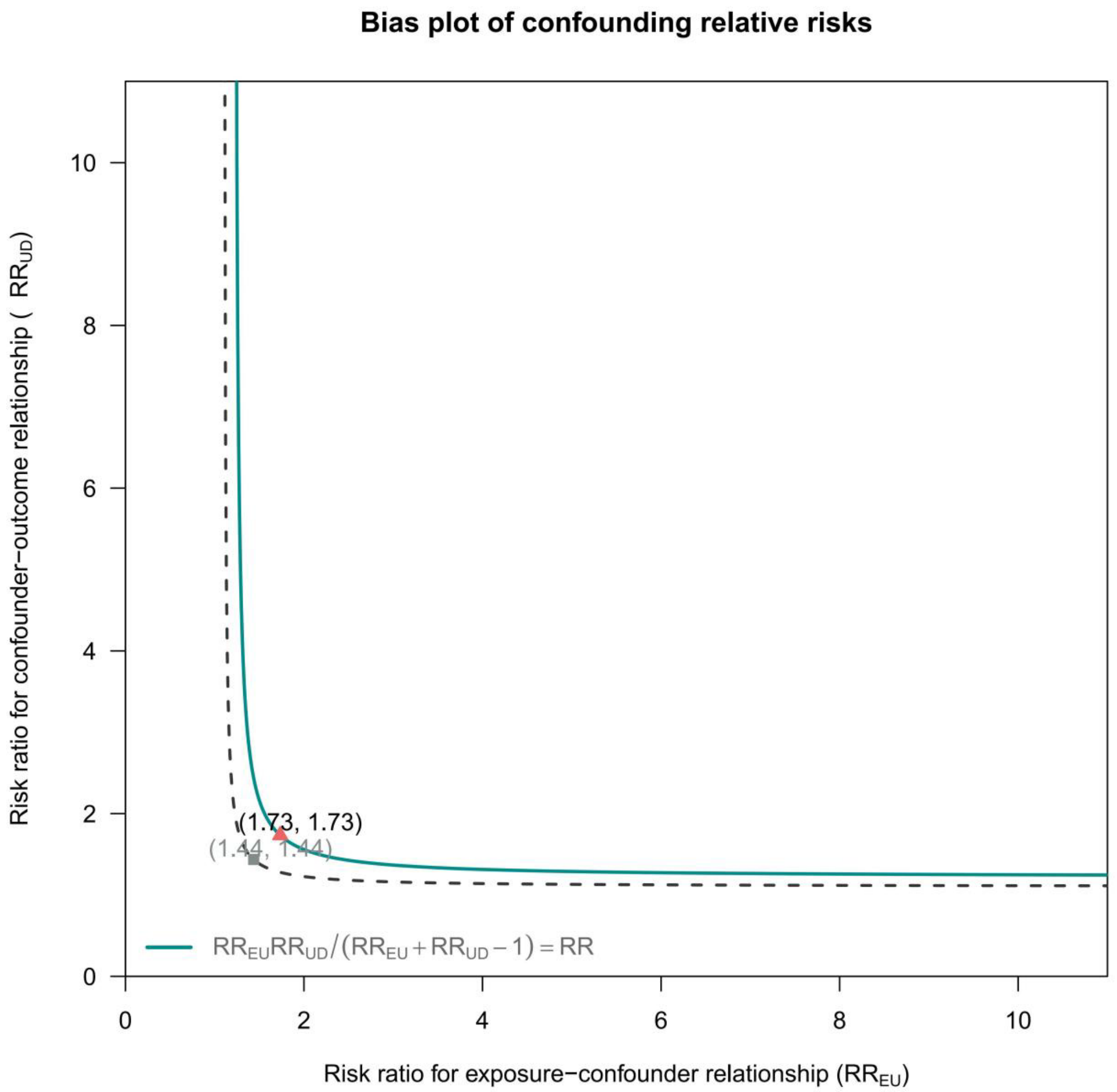

E-value analysis was performed to assess the potential impact of unmeasured confounding factors on the observed association between log_2BAR and 28-day mortality. The bias plot illustrates the calculated E-values to evaluate the robustness of this association. The solid line represents the calculated boundary for the risk ratio of the exposure–confounder relationship (RREU)and the confounder–outcome relationship (RREU).The analysis indicates that the observed association between log_2BAR and 28-day mortality remains robust unless an unmeasured confounder is associated with both log_2BAR and 28-day mortality risk with a magnitude greater than 1.73 (E-value = 1.73). The points (1.73, 1.73) and (1.44, 1.44) are marked in the plot to highlight these sensitivity thresholds. As the strength of a potential unmeasured confounder increases, the curve approaches the reference line for the observed risk

ratio (RR).
