# Supplementary material for: Bridging the gap between biomarkers and bedside decisions: the prognostic value of the blood urea nitrogen-to-albumin ratio in sepsis-associated AKI
Source: Front Nutr. 2026 Aug 13;13:1831995. doi: 10.3389/fnut.2026.1831995 (PMC13518199; doi:10.3389/fnut.2026.1831995)
Supplement: Supplementary file 6 [file Image_6.pdf]

Supplementary Figure 6. Standalone ROC curves of individual clinical scoring systems and log2BAR for predicting 28-day mortality.

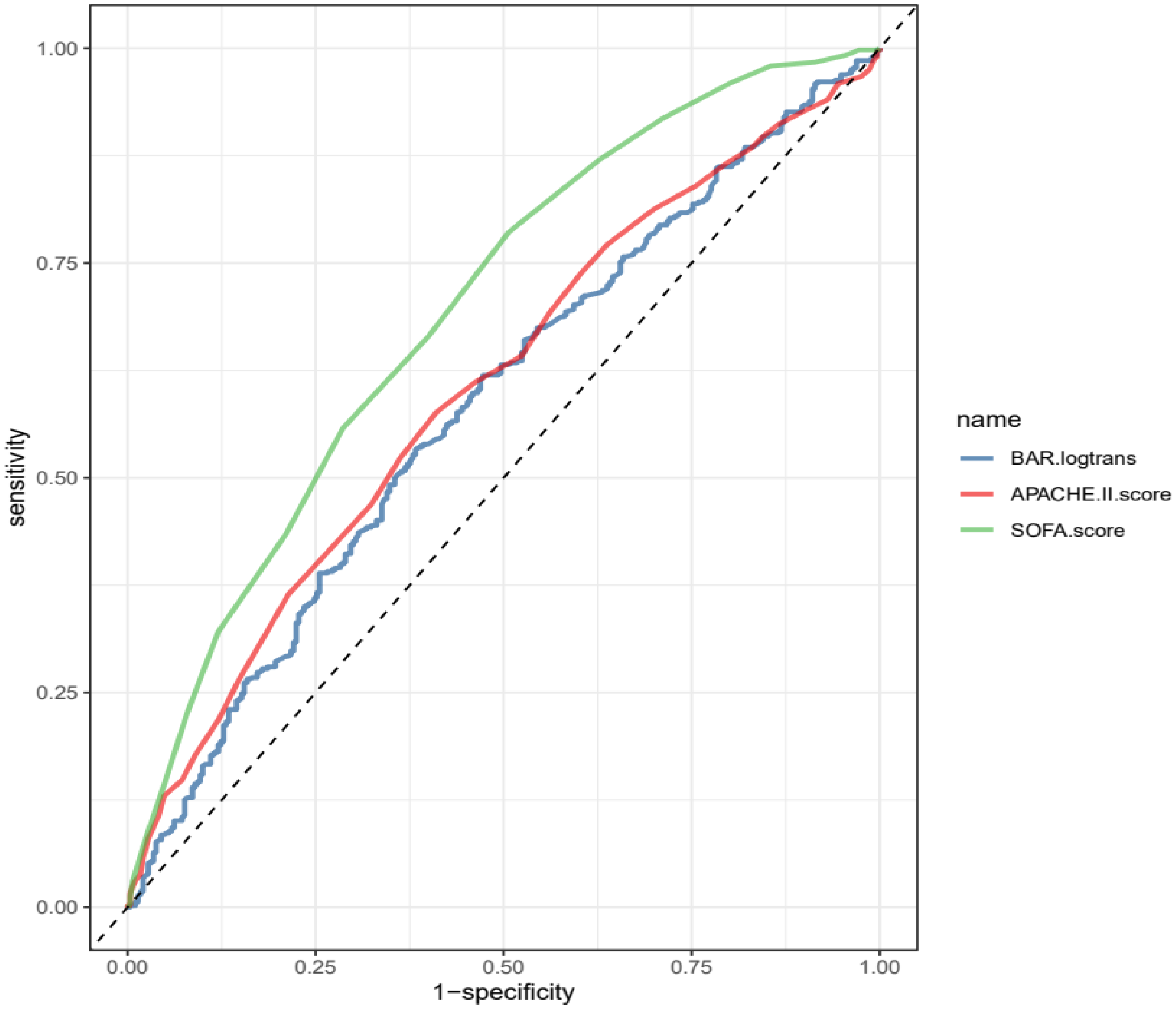

log_2BAR: logarithmically transformed blood urea nitrogen-to-albumin ratio (base 2); SOFA: Sequential Organ Failure Assessment; APACHE II: Acute Physiology and Chronic Health Evaluation II; ROC: receiver operating characteristic; AUC: area under the curve.
