# Supplementary material for: Bridging the gap between biomarkers and bedside decisions: the prognostic value of the blood urea nitrogen-to-albumin ratio in sepsis-associated AKI
Source: Front Nutr. 2026 Aug 13;13:1831995. doi: 10.3389/fnut.2026.1831995 (PMC13518199; doi:10.3389/fnut.2026.1831995)
Supplement: Supplementary file 7 [file Table_1.docx]

Supplementary Table 1. Missing data analysis of clinical variables

| Variable | Valid Samples | Missing Samples | Missing Ratios, % |
| --- | --- | --- | --- |
| Age | 790 | 0 | 0 |
| Albumin | 790 | 0 | 0 |
| APACHE.II.score | 778 | 12 | 1.519 |
| BAR | 790 | 0 | 0 |
| BMI | 774 | 16 | 2.0253 |
| BUN | 790 | 0 | 0 |
| CCI | 790 | 0 | 0 |
| Cerevascular_dis | 787 | 3 | 0.3797 |
| CHF | 790 | 0 | 0 |
| COPD | 790 | 0 | 0 |
| Creatinine | 790 | 0 | 0 |
| CRP | 624 | 166 | 21.0127 |
| Death_28D | 790 | 0 | 0 |
| dementia | 790 | 0 | 0 |
| DM | 789 | 1 | 0.1266 |
| Hemoglobin | 789 | 1 | 0.1266 |
| HTN | 790 | 0 | 0 |
| MAP | 790 | 0 | 0 |
| MI | 790 | 0 | 0 |
| MV_CRRT | 789 | 1 | 0.1266 |
| Phosphate | 760 | 30 | 3.7975 |
| PVD | 790 | 0 | 0 |
| S28TIME | 790 | 0 | 0 |
| Sex | 790 | 0 | 0 |
| SOFA.score | 788 | 2 | 0.2532 |

Abbreviations: APACHE II, Acute Physiology and Chronic Health Evaluation II; BAR, blood urea nitrogen-to-albumin ratio; BMI, body mass index; BUN, blood urea nitrogen; CCI, Charlson comorbidity index; Cerevascular_dis, cerebrovascular disease; CHF, congestive heart failure; COPD, chronic obstructive pulmonary disease; CRP, C-reactive protein; DM, diabetes mellitus; HTN, hypertension; MAP, mean arterial pressure; MI, myocardial infarction; MV_CRRT, mechanical ventilation or continuous renal replacement therapy; PVD, peripheral vascular disease; S28TIME, time to 28-day outcome; SOFA, Sequential Organ Failure Assessment; STATUS 28, 28-day mortality.
