# Supplementary material for: Bridging the gap between biomarkers and bedside decisions: the prognostic value of the blood urea nitrogen-to-albumin ratio in sepsis-associated AKI
Source: Front Nutr. 2026 Aug 13;13:1831995. doi: 10.3389/fnut.2026.1831995 (PMC13518199; doi:10.3389/fnut.2026.1831995)
Supplement: Supplementary file 8 [file Table_2.docx]

Supplementary Table 2. Multivariable Cox regression analysis of the association between log_2_BAR Tertile and 28-day mortality.

| Categories | Model 1 | | Model 2 | | Model 3 | |
| --- | --- | --- | --- | --- | --- | --- |
|  | OR (95%CI) | *P* value | OR (95%CI) | *P* value | OR (95%CI) | *P* value |
| log_2_BAR | 1.23 (1.10~1.37) | <0.001 | 1.23 (1.10~1.37) | <0.001 | 1.33 (1.15~1.53) | <0.001 |
| log_2_BAR Tertile |  |  |  |  |  |  |
| Q1 (<3.96) | 1(Ref) |  | 1(Ref) |  | 1(Ref) |  |
| Q2(3.96-4.72) | 1.14 (0.91~1.42) | 0.267 | 1.13 (0.90~1.41) | 0.301 | 1.13 (0.86~1.5) | 0.371 |
| Q3(>4.72) | 1.55 (1.25~1.92) | <0.001 | 1.54 (1.24~1.92) | <0.001 | 1.74 (1.32~2.3) | <0.001 |
| *P* for trend |  | <0.001 |  | <0.001 |  | <0.001 |

Abbreviations: AKI, acute kidney injury; BAR, blood urea nitrogen-to-albumin ratio; CI, confidence interval; HR, hazard ratio; Ref, reference group.

Note: Cox proportional hazards models were used to estimate hazard ratios (HRs) and 95% confidence intervals (CIs) for 28-day mortality. log_2_BAR was calculated as the base-2 logarithm of the BAR to normalize the data, and HRs represent the risk per doubling of the ratio.

Model 1:Non-adjusted

Model 2:adjusted for Age,Sex

Model 3:adjusted for Age,Sex,HTN,Phosphate,CCI,Creatinine,CRP,SOFA score
