# Supplementary material for: Bridging the gap between biomarkers and bedside decisions: the prognostic value of the blood urea nitrogen-to-albumin ratio in sepsis-associated AKI
Source: Front Nutr. 2026 Aug 13;13:1831995. doi: 10.3389/fnut.2026.1831995 (PMC13518199; doi:10.3389/fnut.2026.1831995)
Supplement: Supplementary file 9 [file Table_3.docx]

Supplementary Table 3. Multivariable Cox regression analysis of the association between log_2_BAR Quintiles and 28-day mortality.

| Categories | Model 1 | | Model 2 | | Model 3 | |
| --- | --- | --- | --- | --- | --- | --- |
|  | OR (95%CI) | *P* value | OR (95%CI) | *P* value | OR (95%CI) | *P* value |
| log_2_BAR Quintiles |  |  |  |  |  |  |
| Q1 (<3.58) | 1(Ref) |  | 1(Ref) |  | 1(Ref) |  |
| Q2(3.58-4.09) | 0.9 (0.67~1.20) | 0.466 | 0.88 (0.66~1.19) | 0.414 | 0.84 (0.59~1.20) | 0.336 |
| Q3(4.09-4.57) | 1.12 (0.84~1.50) | 0.425 | 1.1 (0.82~1.47) | 0.51 | 1.07 (0.75~1.51) | 0.711 |
| Q4(4.57-5.04) | 1.23 (0.93~1.63) | 0.153 | 1.21 (0.91~1.61) | 0.192 | 1.39 (0.98~1.96) | 0.066 |
| Q5(>5.04) | 1.54 (1.16~2.03) | 0.002 | 1.52 (1.15~2.01) | 0.004 | 1.77 (1.24~2.52) | 0.002 |
| *P* for trend |  | <0.001 |  | <0.001 |  | <0.001 |

Abbreviations: AKI, acute kidney injury; BAR, blood urea nitrogen-to-albumin ratio; CI, confidence interval; HR, hazard ratio; Ref, reference group.

Note: Cox proportional hazards models were used to estimate hazard ratios (HRs) and 95% confidence intervals (CIs) for 28-day mortality. log_2_BAR was calculated as the base-2 logarithm of the BAR to normalize the data, and HRs represent the risk per doubling of the ratio.

Model 1:Non-adjusted

Model 2:adjusted for Age,Sex

Model 3:adjusted for Age,Sex,HTN,Phosphate,CCI,Creatinine,CRP,SOFA score
